# Constraints on hand-foot coordination associated with phase dependent modulation of corticospinal excitability during motor imagery

**DOI:** 10.3389/fnhum.2023.1133279

**Published:** 2023-06-30

**Authors:** Kento Nakagawa, Saeko Kawashima, Kazuki Fukuda, Nobuaki Mizuguchi, Tetsuro Muraoka, Kazuyuki Kanosue

**Affiliations:** ^1^Faculty of Sport Sciences, Waseda University, Saitama, Japan; ^2^Research Fellow of Japan Society for the Promotion of Science, Tokyo, Japan; ^3^Research Organization of Science and Technology, Ritsumeikan University, Shiga, Japan; ^4^College of Economics, Nihon University, Tokyo, Japan; ^5^Institute of Health and Sports Science & Medicine, Juntendo University, Chiba, Japan

**Keywords:** interlimb coordination, motor imagery, directional constraint, mental chronometry, MEP

## Abstract

Interlimb coordination involving cyclical movements of hand and foot in the sagittal plane is more difficult when the limbs move in opposite directions compared with the same direction (directional constraint). Here we first investigated whether the directional constraint on hand-foot coordination exists in motor imagery (imagined motion). Participants performed 10 cyclic coordinated movements of right wrist flexion-extension and right ankle dorsiflexion-plantarflexion as quickly and precisely as possible, in the following three conditions; (1) actual movements of the two limbs, (2) imaginary movements of the two limbs, and (3) actual movement of one limb combined with imaginary movement of the other limb. Each condition was performed under two directions; the same and the opposite direction. Task execution duration was measured as the time between the first and second press of a button by the participants. The opposite directional movement took a significantly longer time than did the same directional movement, irrespective of the condition type. This suggests that directional constraint of hand-foot coordination occurs even in motor imagery without actual motor commands or kinesthetic signals. We secondarily examined whether the corticospinal excitability of wrist muscles is modulated in synchronization with an imaginary foot movement to estimate the neural basis of directional constraint on imaginary hand-foot coordination. The corticospinal excitability of the forearm extensor in resting position increased during dorsiflexion and decreased during plantarflexion similarly in both actual and imaginary foot movements. This corticospinal modulation depending on imaginary movement phase likely produces the directional constraint on the imaginary hand-foot coordination.

## Introduction

**FIGURE 1:**
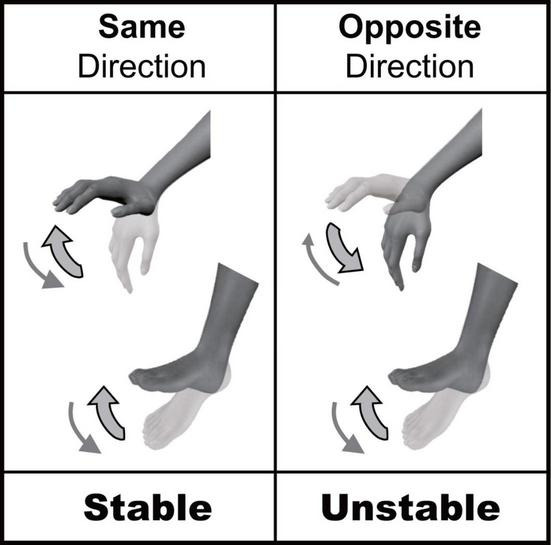
Directional constraint of hand-foot coordination.

There are many opportunities to coordinate multiple limbs in daily life. Several behavioral constraints have been reported to exist in multi-limb coordination. For example, cyclic simultaneous movements of ipsilateral hand and foot are less stable when movements occur in the opposite direction than when they occur in the same direction (Baldissera et al., 1982, 1991; Kelso and Jeka, 1992; Salesse et al., 2005; Muraoka et al., 2013; Nakagawa et al., 2013, 2015); this is called “directional constraint” (Figure 1). The directional constraint of hand-foot coordination in the sagittal plane has been shown to be a robust phenomenon. It does not depend on the presence or absence of visual input, nor the combination of contracted muscles (Salesse et al., 2005; Muraoka et al., 2013). Thus, the stability of hand-foot coordination is rigidly constrained by the movement direction. We have previously shown that directional constraint appeared even when the voluntary movement of one limb (hand) was coordinated with the passive movement of the other limb (ankle) (Nakagawa et al., 2013, 2015). This indicated that directional constraint could appear even without voluntary movements of both limbs. Although we focused on how the afferent information from two limbs is processed in these previous studies, it is still unclear whether the actual afferent information from two limbs is necessary for producing directional constraint. To answer this question, in the present study we used motor imagery that shares neural circuits with actual movement (Hanakawa et al., 2008; Guillot and Collet, 2010). Comparison of “actual movement” and “motor imagery” has been widely used in cognitive psychology and neurophysiology to investigate the control mechanisms of voluntary movements other than actual motor commands and afferent signals (Nair et al., 2003; Oullier et al., 2005; Guillot and Collet, 2010), because motor imagery is considered to be one form of actual movement that occurs without motor commands to muscles and the resultant afferent signals from peripheral receptors. The first aim of this study was to test whether directional constraint appears even during motor imagery of hand-foot coordination (Experiment 1).

To test the above research question, we need to compare the performances between the opposite and same directional movements in imagery. Although motor imagery cannot be investigated by analyzing kinematic information, other aspects of motor imagery can be analyzed. As one such technique, “mental chronometry” which is defined as measurements of the execution time required to complete motor imagery of a task has been used to evaluate how motor imagery is conducted (Guillot and Collet, 2005b). Generally, the time spent for imagery tends to be longer when the imagery is more difficult (Decety and Jeannerod, 1995; Guillot and Collet, 2005b; Mizuguchi et al., 2012a,^2015^; Dahm and Rieger, 2016b). For example, the duration of imaginary drawing of a cube becomes longer when the amplitude of the line of tracing the cube is small compared to large amplitude (Decety and Michel, 1989). Similarly, the time spent imagining walking through gates is inversely associated with the width of the gates (Decety and Jeannerod, 1995). These results are in accordance with Fitts’s law (i.e., speed-accuracy trade-off) and suggest the existence of constraints even in the context of motor imagery. Therefore, the constraints in relation to motor imagery can be evaluated with mental chronometry task because tasks involving challenging imagery take longer to complete than an easy imagery. Mental chronometry has been widely utilized for evaluating motor imagery and is accepted as a reliable method (Malouin et al., 2008). However, since there is no guarantee that participants correctly execute an imaginary movement as instructed, executing only mental chronometry would lack objectivity. Therefore, we compared the task of purely imaginary movements to the task of combining actual movement in one limb with imaginary movement in another limb. Our hypotheses in Experiment 1 were as follows: (1) directional constraint appears in imaginary coordinated hand and foot movements, and (2) when the imaginary movement of a limb is coordinated with the actual movement of the other limb, imaginary limb movement will affect the kinematics of the actual movement of the other limb, and its effect will depend on movement direction.

The second aim of this study is to examine the possible neural basis of directional constraint on hand-foot coordination, including motor imagery. Previous studies shows that corticospinal excitability of a resting upper limb muscle is modulated depending on the phase (see Supplementary Figure 1) of cyclic movement in the ipsilateral lower limb in a way that facilitates the same directional movement of the ipsilateral hand and foot (Baldissera et al., 2002; Cerri et al., 2003; Borroni et al., 2004). To be specific, corticospinal excitability of the wrist extensor muscle with the forearm in the prone position is facilitated during dorsiflexion (DF) of the ipsilateral ankle, while it is suppressed during plantar flexion (PF), and vice versa for the wrist flexor. Since the magnitude of this corticospinal modulation is correlated with the magnitude of the directional constraint on hand-foot coordination (McIntyre-Robinson and Byblow, 2013), the modulation of corticospinal excitability is believed to be the neural mechanism of directional constraint. Therefore, a similar phenomenon could appear, i.e., modulation of corticospinal excitability in one limb depending on the phase of imaginary cyclic movement in the other limb. Thus, using transcranial magnetic stimulation (TMS), we tested the hypothesis that corticospinal excitability of the forearm muscle is modulated by the phase of imaginary movement of the foot, thereby facilitating directional constraint (Experiment 2).

## Materials and methods

The purpose of the Experiment 1 was to test whether directional constraint of hand-foot coordination appears even during motor imagery. To do this, we evaluated (1) the task duration of imaginary coordinated movements, (2) vividness of motor imagery, and (3) variability of kinematic movements.

In the Experiment 2, for examining the neural basis of the directional constraint of hand-foot coordination during motor imagery, we tested the hypothesis that corticospinal excitability of the forearm muscle is modulated by the phase of imaginary movement of the foot.

### Experiment 1: hand-foot coordination including motor imagery

#### Participants and experimental settings

Based on our preliminary experimental data (*N* = 3) relating to the difference in duration of imaginary hand-foot coordination depending on movement direction, the sample size was calculated using an obtained effect size [*d* = 1.33; α-level: 0.05; power (1-β error probability): 0.95]. As a result, calculated necessary sample size was 10.

Five males and five females (22 ± 1 years., mean age ± SD) participated in this experiment. We confirmed that all participants were right handed/footed by self-report. Before the experiment, written informed consent was obtained from all participants. The present study was approved by the Human Research Ethics Committee of Waseda University.

Participants sat comfortably in a chair with the right forearm fixed in the prone position on an armrest. The foot was also in the air because the seat was at higher position. Hand and foot could be moved in the sagittal plane. To measure the time required to execute the task, an electrical switch operated by pressing a button was held in the participant’s left hand. When the button was pressed, the output of the switch was transmitted to a computer. The angular displacements of right wrist and right ankle were measured using electrical goniometers (SG150, Biometrics, UK). The joint signal was low-pass filtered with a cutoff frequency of 10 Hz. Additionally, electromyographic (EMG) activities were recorded from the right extensor carpi radialis (ECR), flexor carpi radialis (FCR), tibialis anterior (TA), and soleus (Sol) muscles with disposable Ag-AgCl surface electrodes placed over the belly of the muscles. Before the electrodes were attached, the involved area of skin was shaved and rubbed with alcohol-soaked gauze to reduce inter-electrode impedance. The EMG signals were amplified by an amplifier (MEB-2216, Nihon Kohden, Japan) and filtered with a band pass of 5–500 Hz. All signals were transmitted and converted by an A/D converter (Power lab 16/30, AD Instruments, Japan) at 1000 Hz and stored on a computer.

#### Task

To evaluate the directional constraint of hand-foot coordination (i.e., differences in performance depending on the movement direction) in the context of motor imagery, the main task was motor imagery related to the periodic coordination of hand and foot movements in the sagittal plane (Imagery + Imagery: ImIm). As explained in the Introduction, the performance of imaginary movements could be evaluated on the basis of the time spent on the mental chronometry task. As an additional performance evaluation, subjective vividness was also reported by the participants. Additionally, to strengthen the results for imaginary hand-foot coordination derived from the mental chronometry task, we included a condition involving the coordination of two actual limb movements (Execution + Execution: ExEx) and a condition involving the coordination of actual and imaginary limb movements (Execution + Imagery: ExIm, and Imagery + Execution: ImEx). In these conditions involving at least one actual movement, we measured the kinematics of the actual hand/foot movements, and the difference (error) between the button-pressing time reported by participants with and the time of the actual hand/foot movements.

**FIGURE 2:**
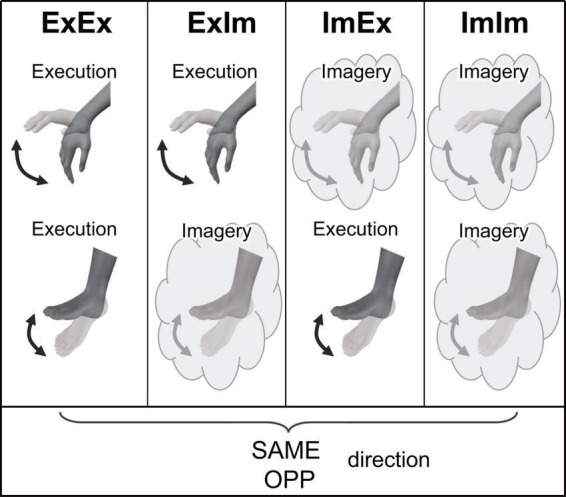
Experimental tasks in Experiment 1. ExEx: coordination of actual movements of both hand and foot, ExIm: coordination of the actual hand movement and imaginary foot movement, ImEx: coordination of imaginary hand movement and actual foot movement, ImIm: coordination of the imaginary movements of both hand and foot. In the four conditions, the same (SAME) and opposite (OPP) directional movements were conducted.

Participants performed eight tasks that were composed of two directions [the same directional movement (SAME) and the opposite directional movement (OPP)] and four conditions (Figure 2). The four conditions differed in the combination of limbs that performed actual execution (Ex) or motor imagery (Im) from the first-person perspective: (1) coordination of actual movements of both hand and foot (ExEx), (2) coordination of the actual hand movement and imaginary foot movement (ExIm), (3) coordination of imaginary hand movement and actual foot movement (ImEx), and (4) coordination of the imaginary movements of both hand and foot (ImIm). The aim of setting the ExIm and ImEx tasks was to reinforce the validity of evaluation by the mental chronometry. That is, these tasks including both actual and imagery movements can be analyzed by mental chronometry and kinematics. We can evaluate the performance of coordinated movements from kinematics too. In each task, participants were asked to perform 10 cyclical movements (i.e., 10 wrist flexion and extension, and ankle dorsiflexion and plantarflexion, movements) in the sagittal plane as fast as possible with their eyes closed. In addition to task speed, we instructed participants to perform each task precisely to avoid phase transition and move their wrist and ankle with their comfortable range and effort of strength. In motor imagery, we asked participants to perform kinesthetic imagery from a first-person perspective.

Before the trial, participants practiced all the tasks. Each task was performed five times. Task conduction was not blocked but randomization was applied in each trial. In each trial, participants pressed a button with their left hand at the start and pressed it again when the task was finished. If the behavioral or imaginary pattern was changed (e.g., transition from the OPP direction to the SAME direction) during the task, participants were asked to correct it. Participants were allowed to take a rest *ad libitum*.

#### Data analysis

The duration of each task was measured as the difference between start and finish times of the task. We also assessed the imagery vividness (Guillot and Collet, 2005a) of each task (except for ExEx) after the experiment by asking participants: “How vivid was your image?” using a 5-point scale (1 = “I could not form an image at all” to 5 = “I formed a vivid image”). The vividness of motor imagery is related to the difficulty of the imaginary motor task (Decety and Lindgren, 1991; Roberts et al., 2008).

To evaluate the stability of actual task movements we analyzed the wrist and ankle joint angles as kinematic information, and calculated the relative phase between the hand and foot movements (only in ExEx), duration of one cycle for each joint. To calculate the relative phase for each cycle, we used the following formula; Φ*_*hf*_* = *360*°*(t_*f,i*_–t_*h,i*_)/(t_*f,i+*1_–t_*f,i*_)*, where *t*_*h,i*_ and *t*_*f,i*_ indicate the time of the *i* th peak extension of the wrist and ankle, respectively (Carson et al., 1995; Ridderikhoff et al., 2005; Volman et al., 2006). We calculated the standard deviation (SD) of relative phase in the ExEx condition as the fluctuation of two coordinated limb movements, and the coefficient of variation (CV) of the cycle duration of each movement was calculated as the fluctuation of one limb’s movement. In addition to the movement stability, the absolute error (AE) of the relative phase for the ExEx task was calculated by averaging the absolute errors in one trial to the target relative phase (SAME: 0°, OPP: 180°).

Additionally, for evaluating the accuracy of task duration as reported by pressing a button using mental chronometry we calculated the time difference between the reported task duration (time between the first and second press of the button) and the duration of 10 cycles of active limb movement in the ExIm and ImEx conditions.

For EMG analysis, the root mean square (RMS) value during the trial (between the first and second press of the button) and in the rest condition in the ECR, FCR, TA, and Sol muscles was calculated. In particular, the RMS in the ECR and FCR muscles was calculated for the ImEx and ImIm conditions. Meanwhile, the RMS in the TA and Sol muscles was calculated for the ExIm and ImIm conditions. To check whether muscle activity was observed during imaginary movements, the RMS value during the imaginary task was compared with RMS during the rest condition.

#### Statistical analysis

Prior to performing parametric statistical analyses, the normality of the data was analyzed by the Shapiro–Wilk test, with the exception of the subjective vividness scores; these data were analyzed by a non-parametric test without an *a priori* normality assessment because of their Likert scale format. We found that the following data did not have a normal distribution: task duration for ImIm_SAME (*p* = 0.03), SD of the relative phase for ExEx_SAME (*p* = 0.04), AE of the relative phase for ExEx_SAME (*p* = 0.04), and the CV of cycle duration for ExIm_SAME (*p* = 0.007). For these data, non-parametric Wilcoxon tests with Holm correction were used to compare the OPP and SAME directions for each index. For normally distributed datasets, the OPP and SAME directions were compared for each condition or index using paired *t*-tests. For electromyographic (EMG) analysis, we compared the RMS value between the imaginary movement and the rest condition. The *p*-value is represented as a corrected value in the section “Results.” The level of significance for all tests was set to *p* = 0.05 in all analyses.

### Experiment 2: neural basis of the directional constraint in motor imagery

#### Experimental setup and apparatus

Based on our preliminary experimental data (*N* = 3) relating to the difference in ECR MEP amplitude depending on imaginary ankle movement phase, we calculated an estimated sample size using an obtained effect size [*d* = 1.23 (α-level: 0.05; power (1-β error probability): 0.95)]. As a result, calculated necessary sample size was 10.

Eight males and two females (22 ± 2 years.) participated in this experiment. Two of them participated in Study 1 too. All participants were right handed/footed according to self-report measures. They sat in a chair with their right forearm on an armrest and fixed in a prone position. The foot was also in the air because the seat was at higher position. Angular displacement of the right ankle was measured by the same setting to the Experiment 1. The EMG signals were measured by the same setting to the Experiment 1. For recording MEP, they were filtered with a band pass of 5–1500 Hz which has been used in previous studies (Mizuguchi et al., 2011; Kato et al., 2016) to reduce low and high frequency noise. All signals were transmitted and converted by an A/D converter (Power lab 16/30, AD Instruments, Japan) at 4000 Hz and stored on a computer. As the Experiment 2 needs to analyze the characteristics of evoked potential, EMG signals were recorded at higher sampling rate (4000 Hz) compared to the Experiment 1 (1000 Hz) (Groppa et al., 2012).

Single-pulse transcranial magnetic stimulation (TMS) was applied using a figure-eight coil (110 mm diameter in each loop) connected to a magnetic stimulator (Magstim 200, Magstim, UK). The coil was positioned on the left primary motor cortex (M1) area of the forearm muscle. The coil was placed tangentially to the scalp over the primary motor cortex with the handle pointing backward and 45° away from the midline, which induces current flow in the postero-anterior direction (Van den Berg et al., 2011). The coil was moved in small increments to determine the scalp position where the largest motor evoked potentials (MEPs) in the right ECR were elicited with the minimum stimulation intensity, i.e., the “hotspot.” In addition, after a rest period, we confirmed that the stimulus location was not altered by checking whether the MEP amplitudes changed in the resting state immediately before the experiment restarted. Resting motor threshold was defined as the lowest TMS intensity that elicited more than five ECR MEPs greater than 50 μV in 10 stimuli (Rossini et al., 1994). The test TMS intensity was 120% of the resting motor threshold (Kitamura et al., 2012; Kato et al., 2016) (67 ± 7% of the maximal output of the stimulator).

#### Tasks

Participants were asked to perform two tasks: (1) execute a cyclic movement of ankle DF and PF (Ex), and (2) imagine the same movement (Im). Both tasks were performed in the sagittal plane and the forearm muscles remained relaxed. They closed their eyes during both tasks. In the Ex task, participants performed voluntary movements of the right foot at a pace dictated by 2 Hz metronome beats by matching the most dorsi- and plantar-flexed position to the beats. Thus, the movement frequency was 1 Hz (McIntyre-Robinson and Byblow, 2013). In the Im task, participants tried to relax all body parts, and kinesthetically imagined the same movement as they performed in the Ex task with the same metronome beats. The actual or imaginary movements started at the peak of plantarflexed position on the fifth beat of the metronome, and the movement continued until TMS was delivered. Thirty-two trials were performed for each task.

Before the experiment, the participants were asked to perform the same tasks to the beat of the metronome. Based on the difference between the movement timing and metronome beat, we calculated an appropriate interval between metronome beat and TMS for each participant so that TMS was applied the foot reached approximately half of either the DF or PF range of movement (ROM) in every trial (Muraoka et al., 2015). ROM was determined using the peak DF and PF angles; 0% and 100% ROM corresponded to the peak PF and DF, respectively, in calculating the DF phase, while 0% and 100% ROM corresponded to the peak DF and PF, respectively, in calculating the PF phase in the Ex task. TMS was applied during either timing of the DF or PF in a random manner. The obtained MEP data were classified into DF or PF phase. If the stimulus point was in the range of 0–5% or 95–100% ROM, the trials were excluded from the analysis, because these points were recognized to be around the peak DF or PF.

The movement phase could not be objectively determined for the Im task. Therefore, right after a trial, participants were asked to judge the phase of a cycle when the TMS had been delivered, from phase 1 to phase 6 (judged phase 1 = PF peak, judged phase 4 = DF peak) (Supplementary Figure 1). Participants reported the number. Judged phase 2 and 3 are defined as DF, and 5 and 6 as PF. The trials in which participants declared the judged phase to be 1 or 4 were excluded from the analysis. The order of tasks was randomized for each participant. The resting MEPs were measured 10 times before the experiment for normalization of MEPs obtained during the tasks. We also measured background EMG signals in a 50 ms window just before the TMS was delivered. If a single data point accounted for more than 20 μV of the amplitude in the window (threshold of EMG appearance), that trial was excluded from the data analysis (Mizuguchi et al., 2012b).

#### Analysis

Amplitudes of MEPs (peak-to-peak) from the ECR were normalized with respect to MEPs during the resting condition. The normalized MEP data were averaged for each movement phase (DF and PF) and task (Ex and Im). The difference in MEP amplitude between the DF and PF phases was tested by non-parametric Wilcoxon tests for each task because the MEP data in PF phase of Im task was not normally distributed (*p* = 0.02). For the timing of Ex stimulus, a paired *t*-test was performed to compare DF and PF phases. For analyzing background EMG activities, we compared the RMS value between DF and PF phases in each muscle using paired *t*-tests. RMS values in TA and Sol muscles during Im task were also compared by paired *t*-tests. The threshold for statistical significance was set at *p* = 0.05.

## Results

### Experiment 1

#### Duration of task execution

**FIGURE 3:**
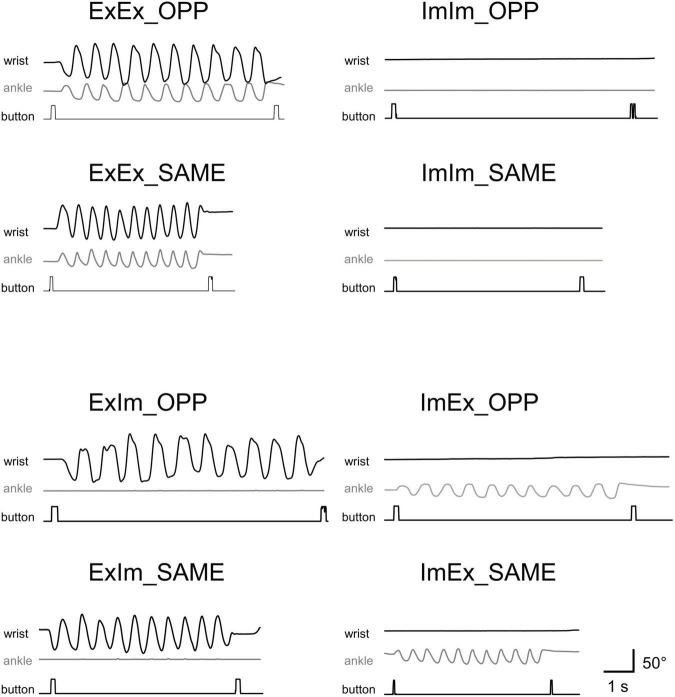
Typical movements in single trials in each task. Wrist and ankle angle changes are represented as black and gray wave forms, respectively. Timing of button-pressing can be detected by square waves.

**FIGURE 4:**
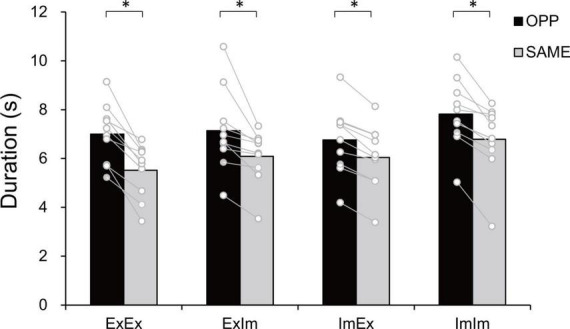
Task duration in the eight tasks. In every condition the opposite (OPP) direction took longer than the same (SAME) direction. An asterisk indicates the significant difference between the OPP and SAME direction. Black and gray bars represent the mean value among participants in each direction. White circles represent the individual datapoint.

Figure 3 shows typical single trials in each task. Figure 4 indicates average task duration for the opposite- (OPP: black bar) and same-directional (SAME: gray bar) movements for the four different conditions. There were significant differences between the OPP and SAME directions in all conditions: the coordination of actual hand and foot movements (ExEx; t[9] = 6.72, *p* < 0.001, *d* = 1.46), coordination of the actual hand movement and imaginary foot movement (ExIm; t[9] = 3.28, *p* = 0.01, *d* = 1.25), coordination of imaginary hand movement and actual foot movement (ImEx; t[9] = 7.90, *p* < 0.001, *d* = 1.23), and coordination of the imaginary hand and foot movements (ImIm; *p* = 0.01, *r* = −0.89). These results indicate that, irrespective of the existence of actual hand or foot movements, the OPP task took longer to perform than the SAME task, as shown in Figure 3.

#### Subjective vividness

**FIGURE 5:**
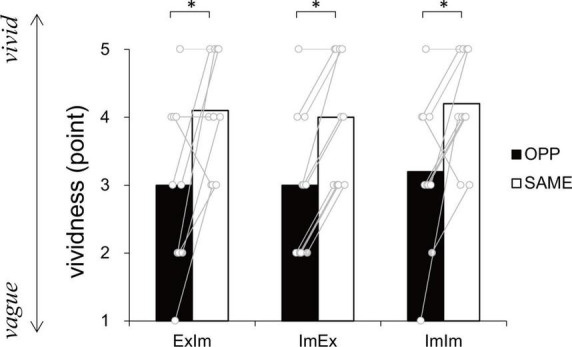
Vividness ratings. In every condition the same (SAME) direction was more vivid than the opposite (OPP) direction. An asterisk indicates the significant difference between the OPP and SAME direction. Black and white bars represent the mean value among participants in each direction. White circles represent the individual datapoint.

Figure 5 shows the subjective vividness in each condition that contains motor imagery. There were significant differences between ExIm_OPP and ExIm_SAME (*p* = 0.03, *r* = −0.68), ImEx_OPP and ImEx_SAME (*p* = 0.01, *r* = −0.91), and ImIm_OPP and ImIm_SAME (*p* = 0.04, *r* = −0.73).

#### Kinematics analysis

**FIGURE 6:**
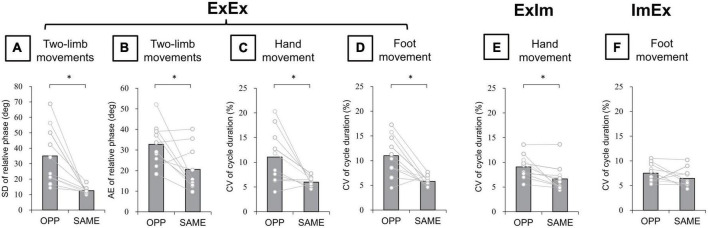
Kinematic data of actual movements. **(A)** SD of relative phase between hand and foot movements. **(B)** Absolute error (AE) of relative phase between hand and foot movements. **(C,D)** CV of hand and foot movement cycle duration in ExEx. **(E)** CV of hand movement cycle duration in ExIm. **(F)** CV of foot movement cycle duration in ExIm. Asterisks indicate the significant difference between the OPP and the SAME direction. An asterisk indicates the significant difference between the OPP and SAME direction. Gray bars represent the mean value among participants. White circles represent the individual datapoint.

In the ExEx condition, standard deviation (SD) of relative phase between wrist and ankle angle was significantly larger in the OPP direction than in the SAME direction (*p* = 0.005, *r* = −0.89) (Figure 6A). Absolute error (AE) of relative phase was also significantly larger in the OPP than the SAME direction (*p* = 0.04, *r* = −0.66) (Figure 6B). Moreover, in each limb movement, the coefficient of variation (CV) of hand (t[9] = 2.94, *p* = 0.02, *d* = 1.30) (Figure 6C) and foot (t[9] = 3.86, *p* = 0.004, *d* = 1.65) (Figure 6D) movement cycle durations were larger in the OPP than in the SAME direction. In the ExIm condition, the CV of cycle duration was larger in the OPP than in the SAME direction (*p* = 0.006, *r* = 0.84) (Figure 6E). However, no significant difference in the CV of foot movement cycle duration between OPP and SAME was observed in the ImEx condition (Figure 6F).

#### Evaluation of accuracy of the reported task duration

**FIGURE 7:**
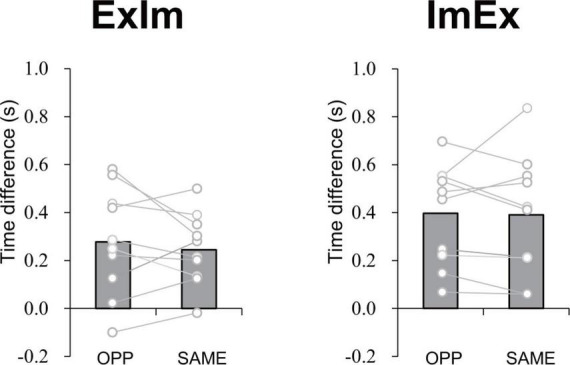
Time difference in elapsed time between the first and second button presses according to the measured duration of active limb movement in each task. The value was calculated by subtracting [reported task duration (time between the first and second press of the button)] from (duration of 10 cycles of active limb movement). No significant difference between OPP and SAME was observed in either ExIm or ImEx. Gray bars represent the mean value among participants. White circles represent the individual datapoint.

The mean time differences between the reported duration and actual duration were 0.28 s for ExIm_OPP, 0.24 s for ExIm_SAME, 0.40 s for ImEx_OPP, and 0.39 s for ImEx_SAME. The time difference between the directions (i.e., OPP vs. SAME) did not differ for each condition (Figure 7).

#### EMG analysis

There was no significant difference in tibialis anterior (TA) or soleus (Sol) muscle activity between the imaginary movements and the rest condition (ExIm_OPP vs. rest, ExIm_SAME vs. rest, ImIm_OPP vs. rest, and ImIm_SAME vs. rest). Likewise, there was no significant difference in extensor carpi radialis (ECR) or flexor carpi radialis (FCR) muscle activity between the imaginary movements and the rest condition (ImEx_OPP vs. rest, ImEx_SAME vs. rest, ImIm_OPP vs. rest, and ImIm_SAME vs. rest). These results suggest that no or trivial muscle activity occurred during imaginary movements.

### Experiment 2

**FIGURE 8:**
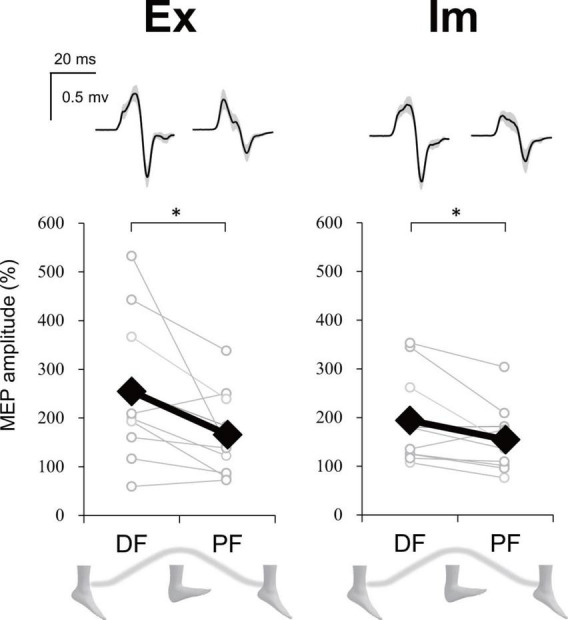
Modulation of extensor carpi radialis (ECR) motor evoked potential (MEP) depending on foot movement phase in Experiment 2. Typical waveforms (mean ± SD) of ECR MEP in each task (Ex: execution, Im: imagery) and ankle movement phase (DF, dorsiflexion; PF, plantar flexion) from a representative participant were shown above. Graphs show the group ECR MEP amplitude data. MEP amplitudes were significantly greater during DF than during PF in both the Ex and Im tasks. Asterisks indicate a significant difference between DF and PF. Black markers of diamond shape represent the mean value among participants. White circles represent the individual datapoint.

For the executing (Ex) task, in total 31.5% of trials were excluded from statistics analysis because the stimulus timing was around the dorsiflexion (DF) or plantarflexion (PF) peak [0–5% or 95–100% range of motion (ROM) or the background electromyographic (EMG)] signal of ECR was above the threshold. For the imaging (Im) task, in total 27.2% of trials were removed because the stimulus timing was reported to be at DF or PF peak (i.e., the trials in which participants judged the phase to be 1 or 4 in imaginary movements) or the background EMG signal of ECR was above the threshold. However, more than 10 trials were able to be analyzed for each phase and task. Upper part in Figure 8 presents the ECR motor evoked potential (MEP) waveform during each ankle movement phase during the Ex and Im tasks from a representative participant. Group data showed that the ECR MEP amplitude during the DF phase was significantly greater than amplitude during the PF phase in the Ex (*p* = 0.02, *r* = −0.73) and Im tasks (*p* = 0.04, *r* = −0.66) (Figure 8).

Stimulus timing during the Ex task was not significantly different between DF and PF (43.5 ± 6.4% ROM and 45.9 ± 7.7% ROM, respectively). The mean value of % ROM indicates that TMS could have been delivered during an intermediate phase in both DF and PF.

For background EMG of ECR and FCR, there were no significant differences in RMS value between DF and PF phases both in Ex (ECR, *p* = 0.81; FCR, *p* = 0.52) and Im (ECR, *p* = 0.14; FCR, *p* = 0.71) tasks. During Im task, RMS value in TA and Sol also did not differ between DF and PF phases (TA, *p* = 0.31; Sol, *p* = 0.71).

## Discussion

We report two main findings: (1) the time spent for executing the opposite directional movement (OPP) tasks was longer than for the same directional movement (SAME) tasks irrespective of whether each limb movement was actual or imaginary, and (2) corticospinal excitability of wrist muscle was modulated depending on the phase of both the imaginary ankle movement and the actual movement in a way that facilitates the same directional movements of the hand and foot.

### Constraints on the actual and the imaginary coordinated movements of two limbs

In the coordination of actual movements of both hand and foot (ExEx), the time it took to complete the task was longer in OPP than in SAME direction. Furthermore, the stability of relative phase between limbs (Figure 6A), the absolute error of relative phase between limb (Figure 6B), and the fluctuation in each limb movement (Figures 6C, D) were also worse for OPP than for SAME. Therefore, it is plausible that the time required to execute a task could be used to evaluate the difference in difficulty between the same and opposite directional hand-foot coordinated movements for imaginary movements as well as for actual movements. In all conditions containing motor imagery, OPP took longer than SAME; this was also seen in the ExEx condition. The mean time differences between task durations and active limb movement duration ranged approximately from 0.2 to 0.4 s (Figure 7) while the differences in task duration between the OPP and SAME ranged approximately from 1 to 1.5 s (Figure 4). Thus, the differences in reported duration between the OPP and SAME tasks cannot be explained by inaccurate button pressing. Furthermore, the difference in task duration corresponded well to the subjective vividness (Figure 5), and also to the single limb movement fluctuation (Figure 6D). Therefore, the mental chronometry results appear to accurately reflect task difficulty, even when the task includes motor imagery.

Our previous studies suggested that sending motor commands to two limbs is not necessary for producing directional constraint on hand-foot coordination; voluntary movement of one limb is sufficient when coordinated with passive movement of the other limb (Nakagawa et al., 2013, 2015). However, whether afferent information from two moving limbs is necessary for directional constraint remains unclear. The result in the condition without any actual movement (i.e., ImIm, involving coordination of imaginary hand and foot movements) suggests that directional constraint occurs even in motor imagery. This expands on our previous findings by indicating that directional constraint of ipsilateral hand-foot coordination not only occurs in the absence of actual motor commands to muscles, but also when there is no afferent input from the limbs. Therefore, the existence of afferent input from limbs or motor commands to muscles is unlikely to be necessary for the directional constraint in hand-foot coordination. Rather, calculating error between limbs may be important for the appearance of the directional constraint regardless of whether the limbs are actually or imaginarily moved. Similarly, in the case of bimanual coordination in the horizontal plane, a constraint on motor imagery similar to what was observed in the current study has been reported (i.e., asymmetrical movements are more unstable than symmetrical movements even if they are performed in motor imagery) (Dahm and Rieger, 2016a,b). On the other hand, Franz and Ramachandran (1998) reported no difference in stability between the bimanual symmetrical and asymmetrical movements when one actively moving hand coordinated with the other hand executing imaginary movement only, which is inconsistent with the results of Dahm and Rieger (2016a,b). Inconsistency among these studies using bimanual coordination could be due to difference in tasks or to movement frequency. For example, Dahm and Rieger (2016a,b) required participants to respond during motor imagery of bimanual movements (i.e., a dual task) which could have increased the cognitive load and may have facilitated the constraint. Meanwhile, the constraint on interlimb coordination during motor imagery might be stronger during hand-foot coordination than during bimanual coordination (Kelso and Jeka, 1992; Nakagawa et al., 2015). Indeed, the cognitive contribution of the constraint on interlimb coordination has been suggested to be more prominent in ipsilateral hand-foot coordination compared with bimanual coordination (Ridderikhoff et al., 2005; Nakagawa et al., 2013, 2015). This may depend on the difference in neural circuits between unilateral and bilateral movements. In ipsilateral hand-foot coordination, signal processing in the primary motor cortex (M1) and in the primary somatosensory cortex mainly occurs in a unilateral hemisphere, which may result in an increased burden on that hemisphere when movements are not in the same direction. Bimanual coordination uses bilateral hemispheres for signal processing.

### Possible neural mechanisms of the constraint on imaginary coordinated movements

A question arises: Why did the opposite directional movements of ipsilateral hand-foot coordination show lower performance than the same directional movements, even in motor imagery? First of all, corticospinal excitability of a resting upper limb muscle is modulated depending on the phase of cyclic movement in the ipsilateral lower limb in a way that facilitates the same directional movements of the ipsilateral hand and foot (Baldissera et al., 2002; Borroni et al., 2004). Since diverging signals might spread in both directions, from upper limb to lower limb as well as from lower limb to upper limb (Muraoka et al., 2015), when the upper limb is moved, a subliminal signal to move the lower limb in the same direction is sent to the M1 foot area. Experiment 2 in the current study showed that the corticospinal excitability of the wrist extensor increased during dorsiflexion (DF) and decreased during plantarflexion (PF), both when the ankle movement was actual and when it was imaginary (Figure 8). Therefore, the same neural modulation as that observed in previous studies (Baldissera et al., 2002; Borroni et al., 2004) appears even in motor imagery. This neural modulation induced by motor imagery would facilitate the directional constraint of even the imaginary coordinated movements and would obstruct the opposite directional movement. Indeed, not only the subliminal neural modulation of wrist muscle, but also the imaginary ankle movement appeared to interfere with hand movement in the ExIm_OPP task (coordination of the actual hand movement and imaginary foot movement in the opposite direction) of Experiment 1 (Figure 6E).

**FIGURE 9:**
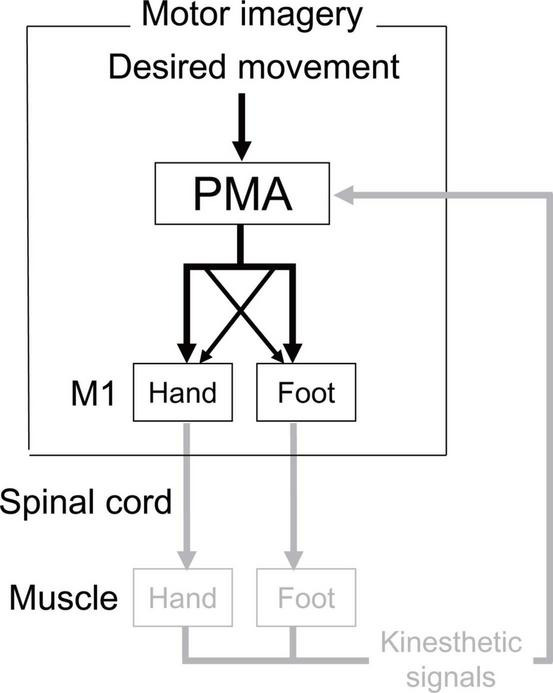
Possible neural circuits of the motor imagery of the hand-foot coordinated movements. In actual execution all these circuits would work, while the circuit expressed in gray would not work in motor imagery. Crossed dotted lines from PMA (premotor area) represents the neural circuits that facilitate the directional constraint. In actual execution all these circuits would work, while the circuit expressed in gray would not work in motor imagery.

Our results for actual and imaginary movements showed a similar trend (Experiment 1: existence of the directional constraint of hand-foot coordination regardless of whether the coordinated movement was actual or imaginary, Experiment 2: possible shared neural circuits facilitating the directional constraint on both actual and imaginary movements). The results suggest that there is, at least in part, a common neural basis for the directional constraint of hand-foot coordination between motor imagery and actual movements. Figure 9 illustrates possible neural circuits of the motor imagery of the hand-foot coordinated movements. As for the possible neural circuits of the directional constraint on hand-foot coordination, Byblow et al. (2007) demonstrated the causal relationship between the corticospinal modulation facilitating directional constraint and the function of the premotor area (PMA). Thus, it is suggested that when a foot is moved, for example, both a suprathreshold signal to the M1 foot area (i.e., a motor command) and a signal, which is usually subliminal, to the M1 hand area are projected from the PMA (represented as the crossed dotted lines in Figure 9). The PMA also plays an important role in motor imagery (Lotze and Halsband, 2006). Combined with the results of Experiment 2 in the current study, during an imaginary movement the PMA might send diverged subliminal signals to the M1 areas innervating the remote limb in the same way as during actual movement. We can speculate that this PMA-M1 neural connection maybe the neural basis for enhanced same directional movement during hand-foot coordination. However, this network that can facilitate same directional movements would be an obstacle when hand and foot movements are coordinated in the opposite direction. Thus, when a movement other than in the same direction is performed, such as in the opposite direction, the network of facilitating the same directional movements should be suppressed. We previously proposed that the network would be suppressed by the supplementary motor area (SMA) (Nakagawa et al., 2016). If the suppression function is insufficient, the movements of the other mode would be involuntarily transferred to the same directional movements. Indeed, in the case of bimanual coordination, when the SMA function is facilitated by brain stimulation, the coordination of the unstable asymmetric movement improves (Carter et al., 2015). In future, further researches should be necessary to test the above-mentioned speculated cortical mechanism of the directional constraint during motor imagery.

In addition to intracortical network, spinal neural network can also influence the interlimb interaction during motor imagery because motor evoked potential (MEP) used in our Experiment 2 reflects summation of cortical and spinal excitability. Indeed, a previous study have shown that spinal reflex excitability of lower leg muscles facilitates by motor imagery of the forearm muscle contraction (Nakagawa et al., 2018). On the other hand, the result of this previous study was that facilitation of spinal reflex excitability by motor imagery of wrist extension was not different between plantarflexor and dorsiflexor muscles, which suggests that interlimb interaction of spinal network during motor imagery would not facilitate the same directional movement, unlike our MEP results. Thus, although it is necessary to experimentally distinguish the cortical and spinal contributions using transcutaneous electrical stimulation or cervicomedullary evoked potentials in future, intracortical network rather than spinal network could possibly contributes to the directional constraint during motor imagery so far.

### Relationship between the motor imagery ability and motor ability

Several studies show that motor imagery ability, as measured by mental rotation tasks, is moderated by motor ability [e.g., Breckenridge et al. (2020)]. In the current study, the subjective vividness of imagery and coordination stability (indicated by the SD of the relative phase) can be considered to reflect motor imagery ability and motor ability, respectively. We also analyzed the relationship between the subjective vividness ratings in the ImIm_OPP condition and SD of the relative phase in the ExEx_OPP condition by Spearman’s rank correlation; no correlation was found. Therefore, it is unlikely that the results obtained in the imagery tasks in the current study were modulated by motor ability. Moreover, considering that previous studies showed that a decrease in motor imagery ability was associated with deterioration of motor ability due to chronic injury or neural disease, and also that our study recruited able-bodied participants, the contradictory results between the previous and current studies are not surprising.

### Clinical significance

We can propose some clinical significances from the results of the present study. It has been already acceptable that motor imagery can facilitate motor learning (Driskell et al., 1994). However, practice of complex multi-limb movements using motor imagery may not overcome the constraint of interlimb coordination, because our results suggested that there is a strong constraint during motor imagery of multi-limb coordination with the neural circuits facilitating the directional constraint. To overcome the constraint during motor imagery, we may need to adjust the imagery so as to avoid making imagery of each limb movement but making them as one integrated movement, as previous studies showed non-perception of each limb movements can overcome the interlimb constraint during actual movements (Mechsner et al., 2001; Kovacs et al., 2010; Muraoka et al., 2016). Therefore, the present study provided important suggestion on the application of practice or learning using motor imagery.

### Limitations

In Experiment 1, we evaluated the elapsed time for cyclic imaginary movements as an indicator of performance in the context of motor imagery. However, we cannot confirm whether the timing of button presses was identical to the start/end times of the motor imagery, which represents a methodological limitation. Thus, directional constraint in the context of motor imagery should be evaluated through multiple indices, where we observed differences in subjective vividness and kinematics, as well as the elapsed time for imaginary movements, between the OPP and SAME directions. As for the subjective vividness, participants reported their imagery vividness in each task after completing all eight tasks. Thus, we cannot deny there was an effect of memory bias on imagery vividness. In the same way, we cannot objectively confirm how the participant’s judgment of the phase of movement cycle during motor imagery was accurate in Experiment 2. Therefore, the results may to be viewed with a caution that memory bias and some errors of subjective evaluation might be involved in motor imagery.

Although we have found that forearm MEP modulated depending on the phase of ankle movement even in motor imagery, it is still unclear whether this modulation shows sinusoidal pattern as shown in the previous studies (Baldissera et al., 2002; Borroni et al., 2004). In future study measurement of MEP at several phases in ankle movement is necessary to answer this question. In Experiment 2, the proportion of gender of the participants was imbalanced (eight male and two female). Gender difference can affect the results. It is ideal to uniform the gender or set the equivalent proportion.

## Conclusion

In conclusion, directional constraint on ipsilateral hand-foot coordination was observed even in tasks involving imaginary movements in terms of duration of task execution, vividness of imagery, and movement fluctuation. These results suggest that a directional constraint on ipsilateral hand-foot coordination could exist in the absence of any actual motor commands or afferent signals from limbs. Furthermore, the corticospinal excitability of the forearm muscle is modulated in synchronization with the phase of imaginary foot movement in a way that facilitates the same directional movements of hand and foot, which could be a neural basis of directional constraint in motor imagery. Neural circuits that produce directional constraints on hand-foot coordination can function without actual movement.

## Data availability statement

The original contributions presented in this study are included in the article/Supplementary material, further inquiries can be directed to the corresponding author.

## Ethics statement

The studies involving human participants were reviewed and approved by the Human Research Ethics Committee of Waseda University. The patients/participants provided their written informed consent to participate in this study.

## Author contributions

KN: conceptualization, methodology, validation, formal analysis, investigation, resources, data curation, writing–original draft, writing-review and editing, visualization, project administration, and funding acquisition. SK and KF: formal analysis and investigation. NM: conceptualization, writing–original draft, and writing-review and editing. TM: writing–original draft and writing-review and editing. KK: conceptualization, resources, writing–original draft, writing-review and editing, project administration, funding acquisition, and supervision. All authors contributed to the article and approved the submitted version.

## Supplementary material

Supplementary Figure 1 Judged phase of the imaginary movement in Experiment 2. Immediately after each trial, participants judged the movement phase (1–6) during which the TMS was delivered.
