# Supplementary figures and images for: Constraints on hand-foot coordination associated with phase dependent modulation of corticospinal excitability during motor imagery
Source: Front Hum Neurosci. 2023 Jun 30;17:1133279. doi: 10.3389/fnhum.2023.1133279 (PMC10348420; doi:10.3389/fnhum.2023.1133279)

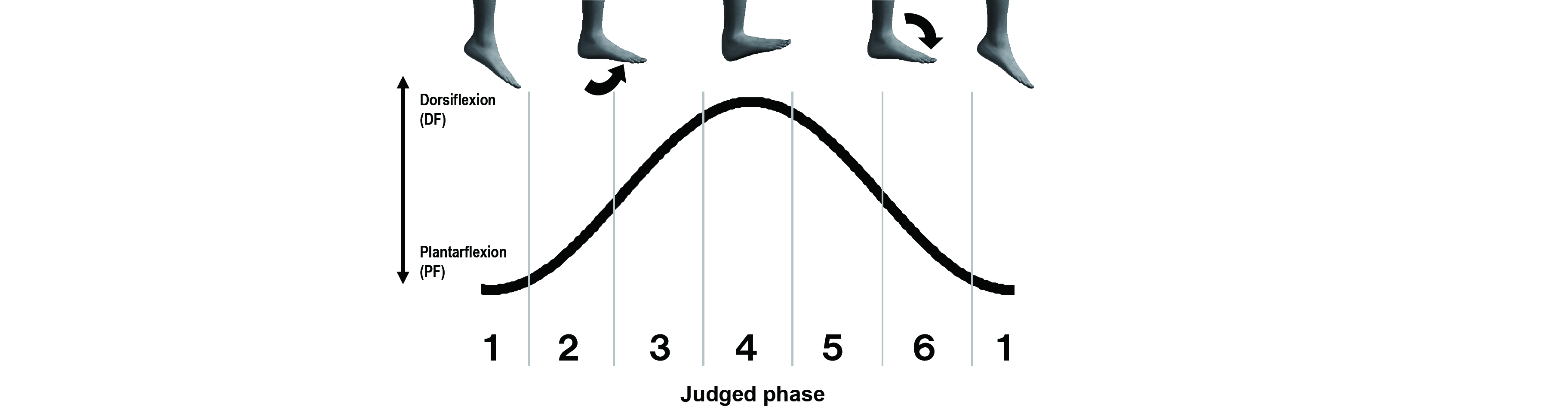

Supplement: Supplementary Figure 1 — Judged phase of the imaginary movement in Experiment 2. Immediately after each trial, participants judged the movement phase (1–6) during which the TMS was delivered. [file Image_1.TIF]
